# Supplementary material for: Diet-induced rewiring of the Wnt gene regulatory network connects aberrant splicing to fatty liver and liver cancer in DIAMOND mice
Source: Sci Rep. 2023 Oct 31;13:18666. doi: 10.1038/s41598-023-45614-1 (PMC10618177; doi:10.1038/s41598-023-45614-1)
Supplement: Supplementary file 1 — Supplementary Information 1. [file 41598_2023_45614_MOESM1_ESM.pdf]

Supplementary information

Diet-induced rewiring of the Wnt gene regulatory network connects aberrant splicing to fatty liver and liver cancer in DIAMOND mice

Ana López-Pérez[1], Silvia Remeseiro[1, 2] & Andreas Hörnblad[1, *]

1. Umeå Centre for Molecular Medicine (UCMM), Umeå University, 90187 Umeå Sweden.
2. Wallenberg Centre for Molecular Medicine (WCMM), Umeå University, 90187 Umeå, Sweden.

* Corresponding author: andreas.hornblad@umu.se

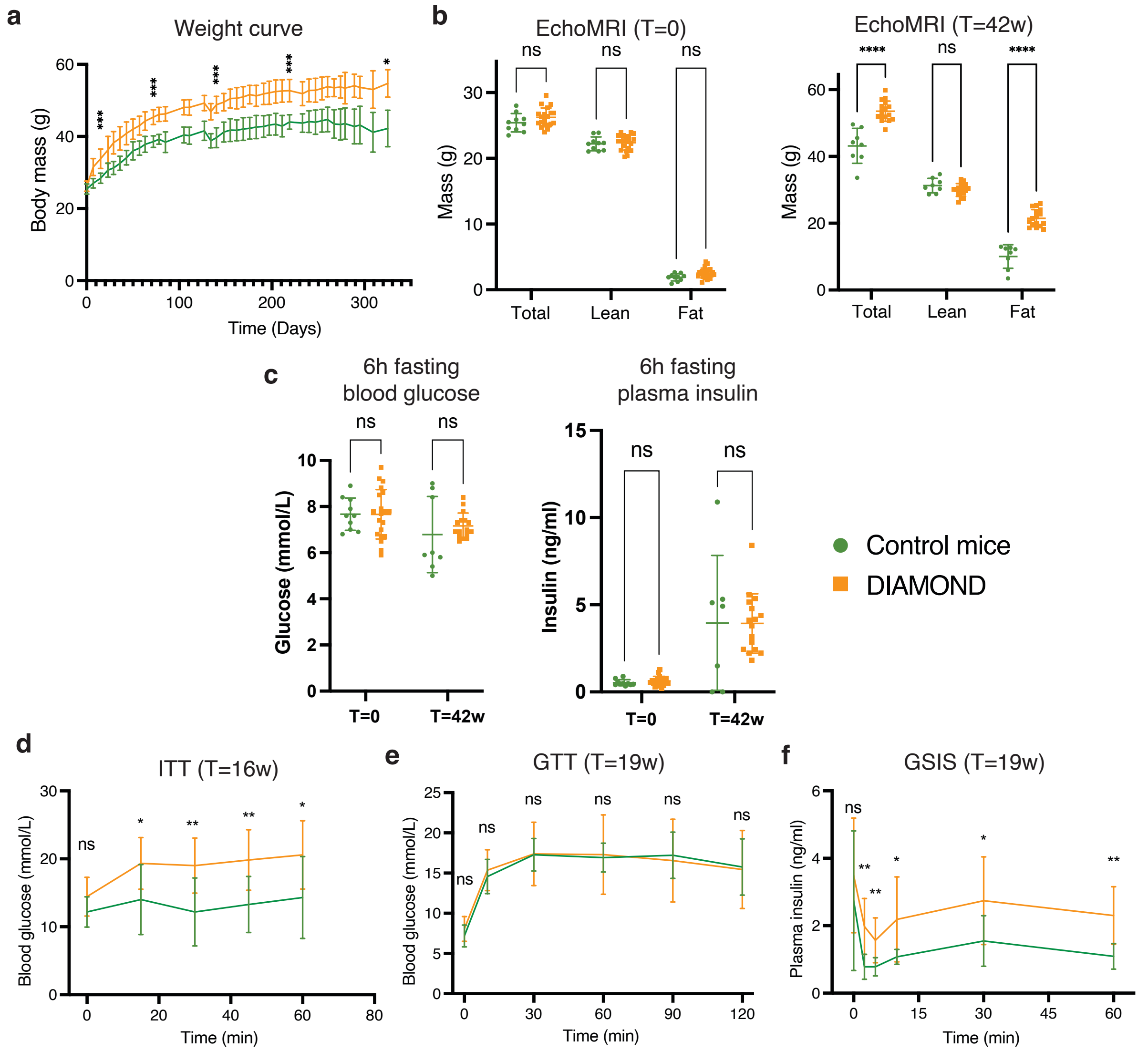

Figure S1. Obesity and insulin resistance in DIAMOND mice. **a**) Body weight change over time in mice fed RD (Control mice, green line) and mice fed WD (DIAMOND mice, orange line). Mean ± SD are indicated. Difference in body mass is statistically significant at all time points from T=7days. For simplification only 5 time points are depicted in the graph. *$p < 0.05$, ***$p < 0.001$. **b**) Body mass composition, **c**) fasted blood glucose (left), and fasted insulin (right) at start of the experiment (T=0) and after 42 weeks of diet (T=42w) for controls (green circles) and DIAMOND (orange squares). Individual data points, mean ± SD are indicated. ****$p < 0.0001$, ns = not significant (Student´s t-test). Blood glucose levels during **d**) Insulin tolerance test (ITT), **e**) Glucose Tolerance Test (GTT), and **f**) insulin plasma profile during Glucose-Stimulate Insulin Secretion test (GSIS) for controls (green) and DIAMOND (orange). Mean ± SD are indicated. *adj.p< 0.05, **adj.p<0.01, ns = not significant (Student´s t-test).

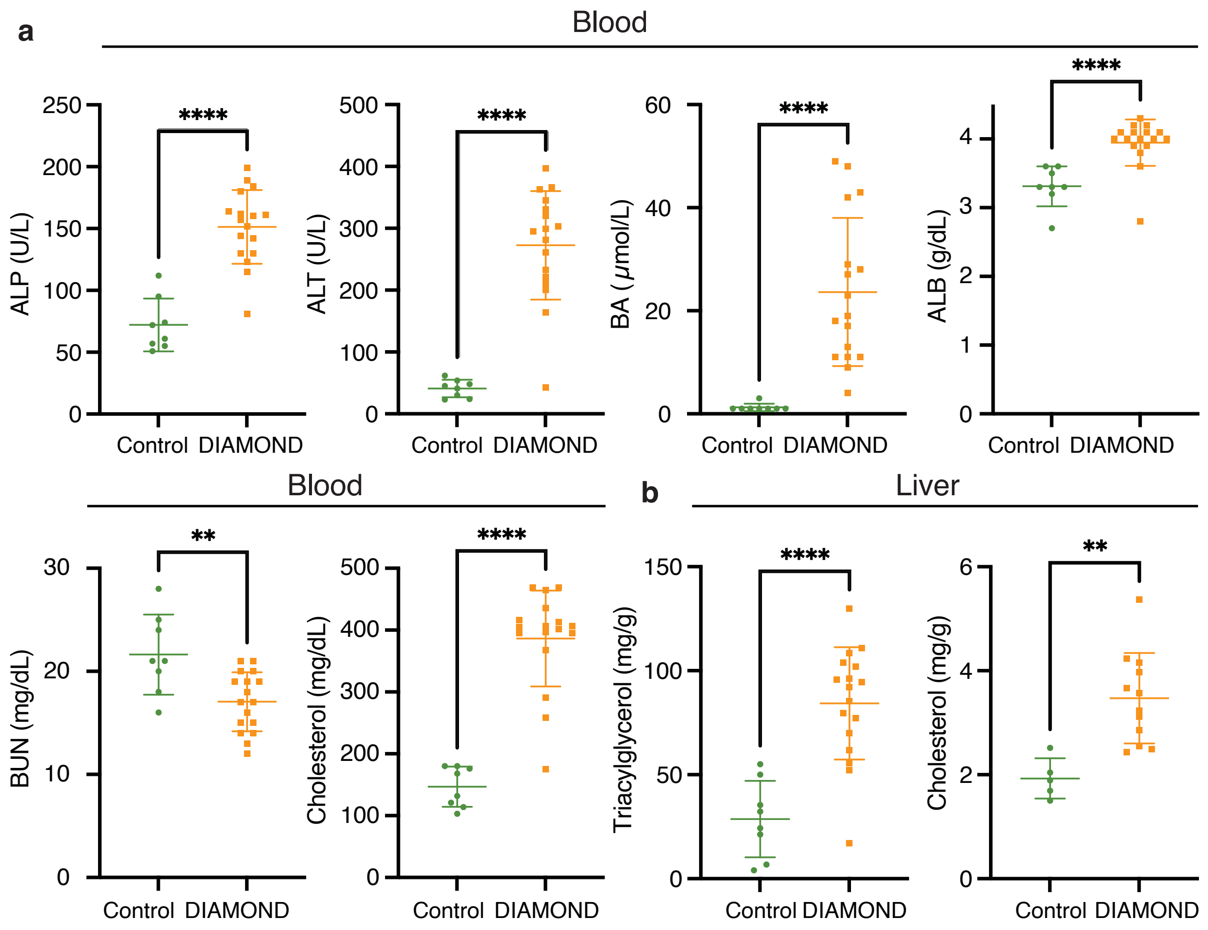

Figure S2. Disturbed liver function and dyslipidemia in DIAMOND mice. **a**) Blood levels of alkaline phosphatase (ALP), aspartate transaminase (ALT), bile acids (BA), albumin (ALB), urea nitrogen (BUN) and cholesterol at T=38w for mice fed regular diet (controls, green) and western diet (DIAMOND, orange). Individual data points, mean ± SD are indicated. **p<0.01, ****p<0.0001 (Student´s t-test for ALP, ALT and BUN, Mann-Whitney test for BA, ALB, and cholesterol). **b**) Liver triacylglycerol and cholesterol levels in the same mice (controls: green, DIAMOND: orange) **p<0.01, ****p<0.0001 (Student´s t-test).

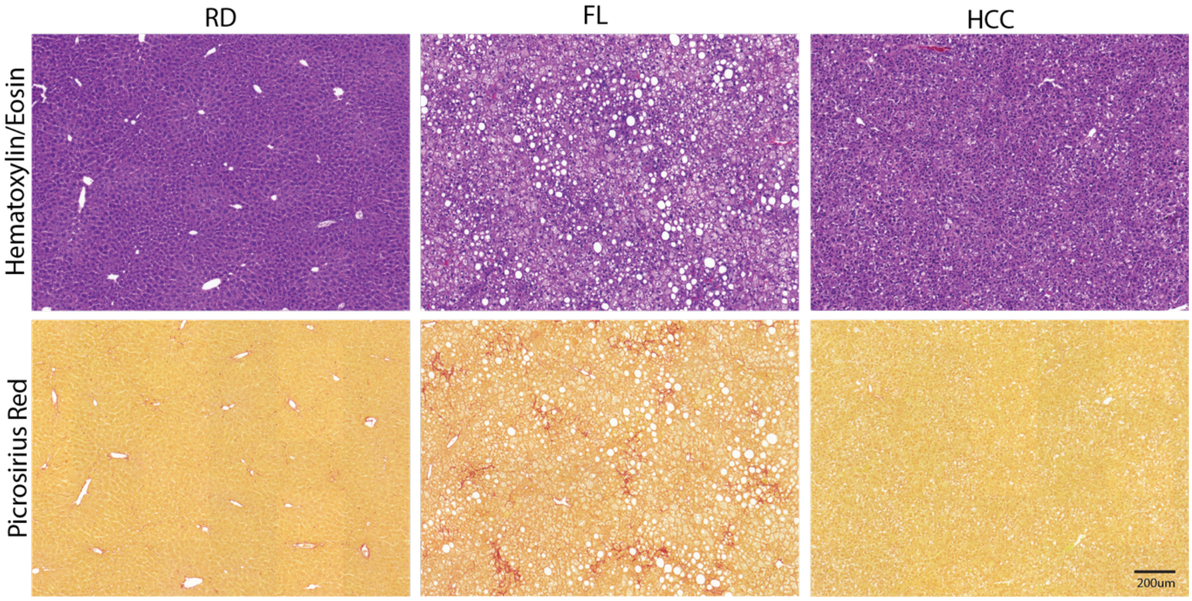

Figure S3. Histology of DIAMOND liver tissue and tumours. Representative photomicrographs of sections from DIAMOND RD livers, FL tissue, and HCC tumours stained for hematoxylin and eosin (upper row) and picrosirius red (lower row). In contrast to fatty liver tissue, the tumours are devoid of fibrosis and lipid accumulation.

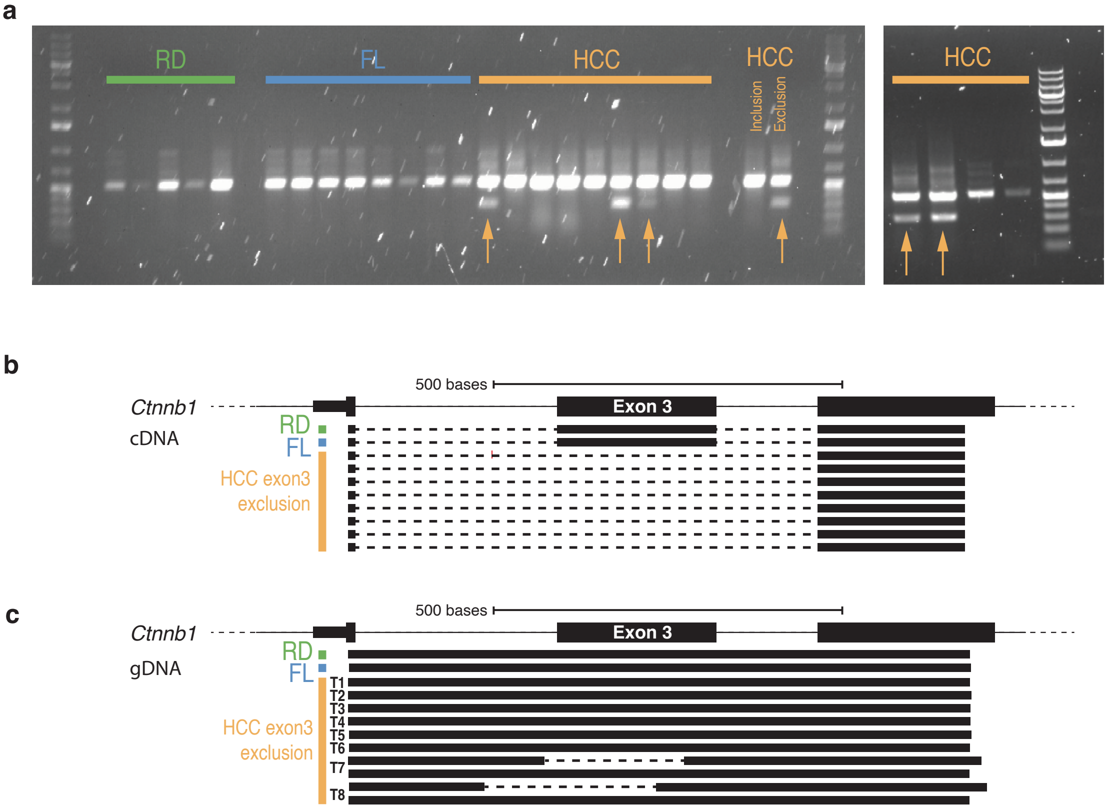

Figure S4. DIAMOND tumours display *Ctnnb1* exon 3 exclusion independent on genomic mutations in the region. **a**) Agarose gel electrophoresis of PCR amplified cDNA from RD liver, FL and HCC tissue. Arrows indicate exon 3 exclusion amplicon. One tumour not excluding exon 3 ("Inclusion") and one tumour with exon 3 exclusion transcripts ("Exclusion") from RNA-seq data were used as controls. **b**) Alignment of Sanger-sequenced *Ctnnb1* transcripts to genomic region. Normal *Ctnnb1* transcripts for 1 RD control liver (green) and 1 FL tissue (blue) are displayed in upper two rows, and exon 3 exclusion transcripts (orange) are displayed below. **c**) Alignment of genomic sequence in the *Ctnnb1* exon 3 region for 1 RD liver (green), 1 FL tissue (blue) and the *Ctnnb1* exon 3 exclusion tumour tissue (orange) from **b**). Note that only two out of 8 exon 3 exclusion tumours have genomic deletions in the region (T7 and T8). For these, both the wild type allele and the deletion are depicted.

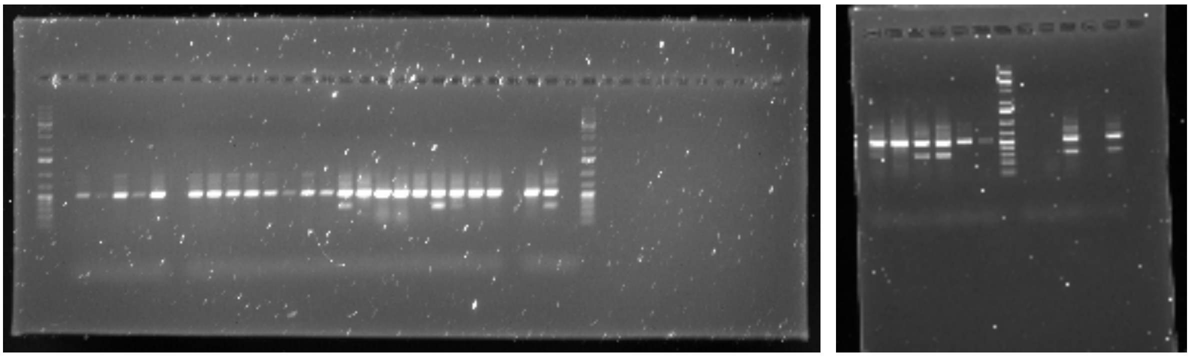

Figure S5. Uncropped images of gels for agarose gel electrophoresis in Figure S4.

Inventory of supplementary tables provided as excel-files

Table S1. Differentially expressed genes between RD, FL and HCC samples with corresponding enriched GO and KEGG terms. Genes were categorised into early, gradual, tumour-specific, fatty liver-specific, and switching.

Table S2. Differentially expressed Wnt-related genes between RD, FL and HCC samples and list of enriched Wnt-related GO term for each gene category. Subset of Table S1 only displaying genes, GO and KEGG terms related to Wnt-signalling.

Table S3. Differential H3K27ac regions in RD, FL and HCC samples and GO and KEGG term enrichment for proximal genes. Regions were categorised into early, gradual, tumour-specific, fatty liver-specific, and switching.

Table S4. Differential H3K27me3 regions in RD, FL and HCC samples and GO and KEGG term enrichment for proximal genes. Regions were categorised into early, gradual, tumour-specific, fatty liver-specific, and switching.

Table S5. Differential Wnt-related H3K27ac regions in RD, FL and HCC samples, with GO and KEGG term enrichment for proximal genes. Subset of Table S3 only displaying genes, GO and KEGG terms related to Wnt-signalling.

Table S6. Differential Wnt-related H3K27me3 regions in RD, FL and HCC samples, and significantly enriched Wnt-related GO and KEGG terms. Subset of Table S4 only displaying genes, GO and KEGG terms related to Wnt-signalling.

Table S7. Complete list of differential alternative splice events between RD, FL and HCC with associated GO and KEGG terms.

Table S8. Table of Wnt-related differential alternative splice events between RD, FL and HCC together with significantly enriched Wnt-related GO terms for each comparison. Subset of Table S7 only displaying genes, GO and KEGG terms related to Wnt-signalling.
